# Variability of Care and Access to Transplantation for Children with Biliary Atresia Who Need a Liver Replacement

**DOI:** 10.3390/jcm11082142

**Published:** 2022-04-12

**Authors:** Jean de Ville de Goyet, Toni Illhardt, Christophe Chardot, Peace N. Dike, Ulrich Baumann, Katherine Brandt, Barbara E. Wildhaber, Mikko Pakarinen, Fabrizio di Francesco, Ekkehard Sturm, Marianna Cornet, Caroline Lemoine, Eva Doreen Pfister, Ana M. Calinescu, Maria Hukkinen, Sanjiv Harpavat, Fabio Tuzzolino, Riccardo Superina

**Affiliations:** 1 Department of Pediatrics, IRCCS-ISMETT (Institute for Scientific-Based Care and Research—Mediterranean Institute for Transplantation and Advanced Specialized Therapies), 90127 Palermo, Italy; fdifrancesco@ismett.edu; 2 Department of General, Visceral and Transplant Surgery, University Hospital Tübingen, 72076 Tübingen, Germany; toni.illhardt@med.uni-tuebingen.de (T.I.); ekkehard.sturm@med.uni-tuebingen.de (E.S.); 3 Division of Pediatric Surgery, Necker Enfants Malades Hospital, 75015 Paris, France; christophe.chardot@nck.aphp.fr (C.C.); marianna.cornet@aphp.fr (M.C.); 4 Division of Pediatric Gastroenterology, Hepatology and Nutrition, Department of Pediatrics, Baylor College of Medicine, Texas Children’s Hospital, Houston, TX 77030, USA; peace.dike@bcm.edu (P.N.D.); harpavat@bcm.edu (S.H.); 5 Division of Pediatric Gastroenterology and Hepatology, Department of Pediatric Kidney, Liver and Metabolic Diseases, Hannover Medical School, 30625 Hannover, Germany; baumann.u@mh-hannover.de (U.B.); pfister.eva-doreen@mh-hannover.de (E.D.P.); 6 Division of Transplant Surgery, Ann & Robert H. Lurie Children’s Hospital of Chicago, Chicago, IL 60611, USA; kabrandt@luriechildrens.org (K.B.); clemoine@luriechildrens.org (C.L.); rsuperina@luriechildrens.org (R.S.); 7 Swiss Pediatric Liver Center, Child and Adolescent Surgery Division, Geneva University Hospitals (HUG), 1205 Geneva, Switzerland; barbara.wildhaber@hcuge.ch (B.E.W.); ana-maria.calinescu@hcuge.ch (A.M.C.); 8 Children’s Hospital, University of Helsinki, 00029 Helsinki, Finland; mikko.pakarinen@hus.fi (M.P.); maria.hukkinen@hus.fi (M.H.); 9 Research Department, IRCCS-ISMETT (Institute for Scientific-Based Care and Research—Mediterranean Institute for Transplantation and Advanced Specialized Therapies), 90127 Palermo, Italy; ftuzzolino@ismett.edu

**Keywords:** biliary atresia, Kasai portoenterostomy, transplant waiting list, pediatric liver transplantation, referral practice, outcome

## Abstract

Background & Aims: Biliary atresia (BA) is the commonest single etiology indication for liver replacement in children. As timely access to liver transplantation (LT) remains challenging for small BA children (with prolonged waiting time being associated with clinical deterioration leading to both preventable pre- and post-transplant morbidity and mortality), the care pathway of BA children in need of LT was analyzed—from diagnosis to LT—with particular attention to referral patterns, timing of referral, waiting list dynamics and need for medical assistance before LT. Methods: International multicentric retrospective study. Intent-to-transplant study analyzing BA children who had indication for LT early in life (aged < 3 years at the time of assessment), over the last 5 years (2016–2020). Clinical and laboratory data of 219 BA children were collected from 8 transplant centers (6 in Europe and 2 in USA). Results: 39 patients underwent primary transplants. Children who underwent Kasai in a specialist -but not transplant- center were older at time of referral and at transplant. At assessment for LT, the vast majority of children already were experiencing complication of cirrhosis, and the majority of children needed medical assistance (nutritional support, hospitalization, transfusion of albumin or blood) while waiting for transplantation. Severe worsening of the clinical condition led to the need for requesting a priority status (i.e., Peld Score exception or similar) for timely graft allocation for 76 children, overall (35%). Conclusions: As LT currently results in BA patient survival exceeding 95% in many expert LT centers, the paradigm for BA management optimization and survival have currently shifted to the pre-LT management. The creation of networks dedicated to the timely referral to a pediatric transplant center and possibly centralization of care should be considered, in combination with implementing all different graft type surgeries in specialist centers (including split and living donor LTs) to achieve timely LT in this vulnerable population.

## 1. Introduction

Biliary atresia (BA) is the most common single indication for liver replacement and transplantation (LT) in children. It is the most common cause of death from liver disease in that age group, and was the indication for 39% of all LT in Europe between 1968 and 2017 [1]. LT is currently proposed as a cure for all children with BA in need of a liver replacement. In the absence of severe comorbidities or contraindications, the risk of these children dying from biliary cirrhosis depends in fact directly on the possibility of obtaining a LT as a timely cure [2,3,4,5,6].

The predictable, progressive and irreversible worsening of their clinical condition during the wait for LT contributes directly to added morbidity and a risk of death in both pre- and post-LT periods [7,8,9,10,11]. Although it is clear and self-evident that a late referral to transplant centers (LTC) and prolonged waiting time for LT are associated with worse outcomes, there are only a few studies analyzing the dynamics of these children’s referrals and of their pathway to LT.

For this study, attention was paid to analyzing the clinical evolution of small children diagnosed with BA and needing LT early in life (<3 years of age), from their diagnosis until LT, and to bring evidence of possible determinants for a successful path to LT (intent-to-transplant analysis).

One hypothesis was that children who are referred secondarily to LTC may experience some delay of assessment and LT (and possible increased morbidity). The analysis aimed at comparing patterns of referral and, in particular, comparing patients who were managed outside an LTC initially, with those who were immediately referred at diagnosis and managed in the center that eventually offered LT. Particular attention was paid to the timing of referral, waiting list dynamics and the need for medical assistance before LT.

## 2. Methods

This study was an international multicentric retrospective analysis. The study concept was generated spontaneously during brainstorming for future research projects on the BA theme, within the BARD association (BA-Related Disorders—http://www.bard-online.com, accessed on 10 March 2022). Eight centers (six in Europe and two in the USA) collaborated for the study.

### 2.1. Study Design and Analysis Plan

Since medical care, transplant medicine and surgery have evolved rapidly, and because liver graft availability/use has varied significantly in the last decades, only the recent and limited period of time (recent five consecutive years) was analyzed. This allowed for analysis of the very current health pathways and ability to further propose methods for improvement in the near future.

Since the reason for liver replacement in BA patients varies with the age, with rapidly progressive liver dysfunction being seen mostly in the younger ones and indications in older ones are mostly related to chronic cirrhosis and portal hypertension or its collateral effects, it was decided to concentrate only on the younger ones, i.e., less than 3 years old, at assessment/registration on the transplant list.

In view of the liver graft allocation policies and graft type, as a consequence, waiting list dynamics substantially differ between Europe and the USA [12,13]. For this reason, separate analyses were run in these two world areas, in order to both compare the two health systems and provide specific conclusions/recommendations for future care.

As all contributing centers were experts in managing children with liver disease and as the time period covered only the recent years, it was considered that clinical approaches and management protocols were sufficiently homogenous enough over the whole period of the study to exclude analyzing/comparing the quality and type of care proposed in these centers (both for pre- and post-LT care, including immunosuppression protocol and surgical techniques).

### 2.2. Study Population: Inclusion and Exclusion Criteria

All consecutive children with BA managed in the respective centers were enrolled if they fulfilled the following criteria: (i) <3 years of age at assessment for LT, and (ii) assessed and transplanted within the contributing centers between 1 January 2016 and 31 December 2020. For those who had been transplanted, only those with a minimum of 3 months follow-up after LT were included at the time of selection and data gathering.

As the study was an “intent-to-LT” analysis, the endpoints were “transplant” or “death (while waiting for LT)”. Patients who were still waiting on the list at the study closure date were excluded, as well as those who were removed from the list during the study period because of being “too-well” or clinically improving to the point that LT was not recommended. On the contrary, those who died while waiting, and those who were removed from the list because they were considered too sick (i.e., a contraindication to LT), were included in the study group. Lastly, patients who had been assessed in one of the contributing centers but were later transferred to another center were excluded from the analysis performed by the former center.

### 2.3. Data for Analysis

All data were retrospective and retrieved from the patients’ medical and operational records. This was performed once and only for the purpose of the analyses by one of the co-authors of the caring center.

Data and information were collected about: (1) The history of prematurity, comorbidities and associated malformations, poly-malformative atresia or not. (2) The type of initial surgery for BA (no-surgery, laparotomy or Kasai procedure) and the type of center for Kasai ((a) non-liver-expert center (i.e., no multidisciplinary pediatric liver service), (b) liver-expert center (i.e., multidisciplinary pediatric liver service available but no transplant service) and (c) pediatric LT center (LTC including multidisciplinary pediatric liver services). (3) Age at initial operation, age at assessment and age at registration on the transplant list. (4) Clinical condition (weight, ascites or not, nutritional support or not and type, Pediatric End-Stage Liver score (PELD)) at the time of assessment and registration on the list. (5) Waiting time on the list before LT, and clinical evolution while waiting (ascites, albumin or blood transfusions, need for hospitalization). (6) Age, weight, PELD and clinical condition (home, hospital- or intensive care unit (ICU)-bound) at LT. (7) Type of donor (deceased or living donor (LD)) and liver graft type (full-size, reduced, split or living donor). (8) Cause of death before LT, cause of death or graft loss after LT and age at last clinical check.

### 2.4. Data Management and Statistical Analyses

Data were initially analyzed as a whole with comparisons between subgroups, and secondly as follows:

1. To analyze referral pathways and their dynamic, subgroups were defined as per the type of initial BA surgery: (A) no-surgery, (B) explorative laparotomy only (no-Kasai), (C) Kasai procedure performed in non-expert liver center, (D) Kasai procedure performed in expert liver center other than the LTC and (E) Kasai procedure performed in the LTC where the transplant was performed later.
2. As access to LT and waiting time for LT are very different when a candidate for LT is proposed to LD-LT, a second analysis was performed with the same subgrouping method, and comparing all patients who had living donor LT versus all others.

For studying correlations, at the level of the centers: between regional allocation rules, waiting list dynamics, LD-LT use and the proportion of LT using PELD exception (or similar priority) request, one center was excluded because regional allocation was not PELD/MELD-based, but center-driven in an otherwise unique national set-up. For this specific study, all mean values were rounded to the nearest integer.

For the statistical analysis, continuous variables were expressed as mean and standard deviations, or as median and range where appropriate. They were compared with the T-Test, Wilcoxon rank sum test or the Mann–Whitney test, and the ANOVA test, together with Levene’s Test, for assessing the homogeneity of variance between the groups. The categorical variables were compared by using Fisher’s exact test or the Chi-square test when appropriate. All the data were analyzed by using SAS 9.4 Software.

*p* < 0.05 was considered statistically significant.

## 3. Results

General demographics and results of the analysis are detailed in Table 1, Table 2 and Table 3.

### 3.1. Europe

During the study period, 165 patients were assessed for LT in 6 LTC. Of the 165 children, 11 had a history of prematurity, 25 had malformative polysplenia syndrome and 27 had other comorbidities (Table 1). Of the 165 patients, 136 underwent Kasai porto-enterostomy after the diagnosis of BA (82%) (mean age at Kasai ± SD, 60.3 ± 25.0 days). A detailed analysis is provided in Table 1 and Table 3.

During their waiting time for a LT, the clinical condition of 150/165 children worsened with any (or a combination) of the following problems: increasing ascites (73%), the need for albumin infusion (59%) or blood transfusions (29%), the need for enteral (40%) or parenteral nutrition (PN) support (22%), the need for short (<5 days) or long (>5 days) hospitalizations (25% and 61%, respectively), or the need for recovery in the ICU (13%).

No deaths occurred during their LT waiting time and all the children were finally transplanted.

Of all LT, 120 (72.7%) were performed in children aged less than 1 year at the time of transplant. At the time of the LT, 88 children were at home, while 63 cases were hospital-bound, and another 14 children were in the ICU. LT were performed with full-size livers (*n* = 20), reduced livers (*n* = 5), split liver grafts (*n* = 60) and grafts procured from LD (*n*= 80). Of all 165 children transplanted over a period of 5 years, death occurred after LT in 5 cases (overall patient survival = 97%). Death occurred within the first post-operative trimester in 4/5 cases (graft primary non-function in 2 cases, sepsis in 1 case and pulmonary hypertension in 1 case), or during the second year in another case (cardiac complication).

Comparison of subgroups A to E (as per type of initial surgery at BA diagnosis) evidenced statistical differences between the subgroups for age at assessment, weight at assessment and initial PELD score (Table 1). The children who had primary transplants and those who had the Kasai operation performed within the center where they received the transplant were younger, weighed less and had lower PELD scores at any time of their management course (from assessment to LT) compared to the other groups.

A comparison of those who benefited from LD versus others showed that children in the former group were similar for age but lower in weight, though they were significantly more likely to develop ascites and the management of the ascites required more albumin infusion and was associated with significantly more hospitalization (including in the ICU). Although their waiting time for LT was significantly shorter, those receiving a LD-LT had worsening PELD scores while waiting and had significantly higher PELD scores at LT compared to the latter group. Interestingly, the duration of respiratory assistance after LT, and the length of stay both in the ICU and in hospital overall, were all significantly shorter in the LD group (Table 3).

### 3.2. USA

General demographics and results of the analysis are detailed in Table 2 and Table 3.

In the American phase of the study, 55 patients in 2 LT centers were included. Only 5 (9%) babies were born prematurely. Of the 55, 9 (16.4%) had polysplenia, 45/55 babies underwent a Kasai portoenterostomy, while 10 (18.2%) either underwent no surgery or just an exploration. The mean age at Kasai operation for the American cohort was 60.0 ± 27.2 days.

During the waiting period, many of the children suffered from a deterioration in their condition: 31/55 (56.4%) experienced worsening ascites and 32/55 (58.2%) required albumin infusions during the waiting period. Though at referral for assessment, 28/55 (51%) had enteral support and only 10/55 (18%) had PN, the latter ratio increased to 27/55 (49.1%) during the waiting time (Table 2). Additionally, 27/55 (49.1%) required at least one blood transfusion, and 40/55 (72.7%) required at least one hospital admission of greater than 5 days as well as at least one admission shorter than 5 days (26/55, 47.2%).

One child died before LT: though he was waiting in ICU and had a PELD exception priority, his condition deteriorated to the point where a LT would be contraindicated and he was removed from the list.

Slightly more than half of the 54 transplanted patients (28/54, 52%) received a transplant while waiting at home, and more than a third (19/54, 35.2%) were in hospital but not in the ICU, with 7 (12.7%) being in the ICU at the time of the transplant.

Of the children undergoing a transplant, 36/54 (67%) were less than a year of age. The average age at transplant was 9.8 months (7.6–13 interquartile range).

The distribution of organ types in the American cohort was 30 (56%) whole livers, 16 (30%) split livers, 2 (4%) reduced size and 6 (11%) living donor left lobes or segment II–III grafts.

Most patients (5/6, 83.3%) who received a LD-LT were in hospital, and 1/6 was in the ICU. Only 1 patient (1/6, 16.7%) was at home at the time of the LD transplant, in contrast to the European live donor patients, most of whom (47/80, 58.7%) were at home at the time of the transplant.

Overall survival in the 5-year period was 51/54 (94.4%), including 6/6 surviving in the live donor group and 45/48 (94%) in the deceased donor group.

A comparison between the five groups (A–E) according to where and if they had a Kasai operation demonstrated that the infants who had either no surgery or an exploration alone (groups A and B) were significantly younger at assessment, listing and transplant than all the other groups. This was followed by the patients who had a Kasai operation and the transplant at the same center (group E) compared to those who had a Kasai at a liver-expert center different from the transplant center (group D). The average age at listing was significantly different among groups, including 5.9 ± 1.1 months for group A, 7.2 ± 4.4 months for Group C, 12.4 ± 7.3 months for Group D and 7.3 ± 2.7 for Group E (*p* = 0.026). Similarly, the age at transplantation was younger for groups A and E (8.1 ± 1.8 and 10.9 ± 4.2 months, respectively) compared to groups C and D (13.0 ± 7.2 and 16.2 ± 10.1 months, respectively). Group B was only 4 patients and did not permit statistical comparison.

Differences in the PELD scores at either listing or transplantation did not differ significantly in the five groups, although PELD score at the time of the transplant tended to be higher in groups A and E, but the difference was not significant (Table 1). The waiting time on the list was shorter in groups A and E but the difference was not significant among the five groups. Group C patients had a higher rate of whole liver transplantations than all other groups, but this was a function of the different donor characteristics and referral patterns between the two American centers rather than because of any differences in the patients themselves.

**Table 1:** Europe group: analysis of subgroups as per initial surgery for biliary atresia.

			A	B	C	D	E	*p*
			NO Surgery	Explorative Laparotomy	KASAI in Non-Liver-Expert Centre	KASAI in Liver-Expert Other Centre	KASAI and Transplant in Same Center	
		N (%)	25 (15%)	4 (2%)	44 (27%)	12 (7%)	80 (49%)	
Polysplenia Syndrome		N (%)	5 (20%)	1 (25%)	6 (14%)	2 (17%)	11 (14%)	*0.9126*
Age at Kasai (if kasai)	(Days)	Mean ± SD	-	-	62.0 ± 25	69.0 ± 18.7	58.2 ± 26.0	*0.4499*
At assessment	Age (months)	Mean ± SD	5.8 ± 3.1	6.0 ± 1.4	10.5 ± 7.2	9.0 ± 5.0	5.8 ± 3.9	**0.0068**
	Weight (Kgs)	Mean ± SD	6.6 ± 1.55	6.8 ± 1.2	7.6 ±2.3	8.0 ± 2.0	6.34 ± 1.9	**0.0058**
	PELD score	score	19.4 ± 7.2	24.5 ± 7.55	16.2 ± 8.9	17.5 ± 7.8	11 ± 7.8	**0.0009**
	Presence of ascites	N (%)	20 (80%)	3 (75%)	27 (61%)	8 (67%)	47 (59%)	*0.3943*
	Enteral nutrition support	N (%)	7 (28%)	1 (25%)	17 (39%)	2 (17%)	35 (44%)	*0.3079*
	Parenteral nutrition support	N (%)	-	-	5 (11%)	3 (25%)	12 (15%)	*0.1639*
At registration on list	Age (months)	Mean ± SD	6.6 ± 3.3	8.25 ± 2.2	12.2 ± 7.5	11.7 ± 5.7	6.5 ± 4.2	**0.0012**
	Weight (Kgs)	Mean ± SD	6.8 ± 1.6	7.2 ±0.7	8.1 ± 2.2	8.4 ± 2.8	6.6 ± 1.9	**0.0005**
	PELD score	score	17.8 ± 6.9	20.7 ± 6.8	15.4 ± 8.2	17.9 ± 8.7	11.5 ± 7.8	**0.0005**
Delta Peld score 1	assessment to registration on list	delta	1.6 ± 4.4	3.7 ± 2.4	0.8 ± 3.4	0.9 ± 3.2	1.4 ± 3.8	*0.5916*
While waiting for LT	Worsening ascites	N (%)	20 (80%)	3 (75%)	28 (64%)	10 (83%)	59 (74%)	*0.5196*
	Albumin infusion(s)	N (%)	15 (60%)	2 (50%)	26 (59%)	10 (83%)	45 (57%)	*0.5254*
	Blood transfusion(s)	N (%)	4 (15%)	1 (25%)	13 (30%)	7 (58%)	23 (29%)	*0.1311*
	Enteral nutrition support	N (%)	11 (44%)	1 (25%)	18 (41%)	6 (50%)	30 (38%)	*0.8922*
	parenteral nutrition support	N (%)	3 (12%)	-	13 (30%)	5 (42%)	16 (20%)	*0.1504*
	1 Hospital admission < 5 days	N (%)	3 (12%)	1 (25%)	6 (14%)	-	10 (12%)	*0.6655*
	>1 Hospital admission < 5 days	N (%)	5 (20%)	-	4 (9%)	5 (42%)	8 (10%)	**0.0227**
	1 Hospital admission > 5 days	N (%)	14 (56%)	1 (25%)	19 (43%)	8 (67%)	43 (54%)	*0.4318*
	>1 Hospital admission > 5 days	N (%)	3 (12%)	-	2 (5%)	-	10 (12%)	*0.3952*
	Recovery in ICU	N (%)	1 (4%)	-	1 (2%)	4 (33%)	16 (20%)	**0.0062**
At Liver transplant	Age (months)	Mean ± SD	9.1 ± 6.05	18 ± 21.4	14.6 ± 8.5	13.4 ± 7.1	8.9 ± 6.1	**0.0012**
	Weight (Kgs)	Mean ± SD	7.5 ± 2.1	8.8 ± 2.9	9.0 ± 2.4	8.9 ± 3.2	7.6 ± 2.1	**0.0072**
	PELD score	score	21.6 ± 6.5	24.0 ± 12.1	17.7 ± 10.5	18.7 ± 10.0	14.9 ± 9.7	**0.0184**
Clinical condition at LT	Elective-Home	N (%)	11 (44%)	3 (75%)	29 (66%)	5 (42%)	40 (50%)	*0.1300*
	Hospital-bound	N (%)	13 (52%)	1 (25%)	15 (34%)	5 (42%)	29 (36%)	
	ICU-bound	N (%)	1 (4%)	-	-	2 (16%)	11 (14%)	
Waiting time	Assessment to LT (Days)	Median (p25–p75)	40 (25–72)	34 (26–695)	77 (45–149)	80 (22–157)	71 (34–121)	**0.3530**
Delta PELD score 2	Assessment to LT	Mean ± SD	3.8 ± 9.0	3.2 ± 5.25	2.4 ± 5.4	0.7 ± 7.4	3.4 ± 6.4	*0.6658*
Graft type	Full-size	N (%)	5 (20%)	-	3 (7%)	-	12 (15%)	*0.6108*
	Split liver graft	N (%)	7 (28%)	1 (25%)	16 (36%)	5 (42%)	31 (39%)	
	Reduced liver	N (%)	-	-	1 (2%)	-	4 (5%)	
	Living donor left lobe	N (%)	13 (52%)	3 (75%)	24 (54%)	7 (58%)	33 (41%)	
post-LT recovery	Respiratory assistance (time—days)	Median (p25–p75)	1 (1–5)	0 (0–2)	1 (0–2)	1 (0–7)	2 (0–6)	*0.1415*
	Enteral nutrition support need	N (%)	17 (68%)	-	26 (59%)	8 (67%)	71 (89%)	**<0.0001**
	Parenteral nutrition need	N (%)	19 (76%)	2 (50%)	29 (66%)	8 (67%)	65 (81%)	*0.2565*
	ITU stay (days)	Median (p25–p75)	7 (2–11)	2 (2–2.5)	4.5 (2–15.5)	4.5 (2–20)	6 (3–12)	*0.3665*
	Hospital stay (days)	Median (p25–p75)	27 (22–35)	24.5 (16.5–32.5)	24 (17–34)	30 (20–44.5)	26 (21–35)	*0.5731*
Outcome	Death while waiting	N (%)	-	-	-	-	-	*0.6584*
	Death after LT	N (%)	-	-	1 (2%)	-	4 (5%)	
	Alive and well	N (%)	25 (100%)	4 (100%)	43 (98%)	12 (100%)	76 (95%)	
	Current age of survivors (months)	Mean ± SD	47.5 ± 21.3	46.7 ± 5.3	48.4 ± 18.2	50.3 ± 15.7	38.3 ± 17.7	**0.0157**

Italic and bold: significant values of *p*.

**Table 2:** USA group: analysis of subgroups as per initial surgery for biliary atresia.

			A	B	C	D	E	*p*
			NO Surgery	Explorative Laparotomy	KASAI in Non-Liver-Expert Centre	KASAI in Liver-Expert Other Centre	KASAI and Transplant in Same Center	
		N	6 (11%)	4 (7%)	18 (33%)	5 (9%)	22 (40%)	
Polysplenia Syndrome		N	1 (17%)	-	2 (11%)	-	6 (27%)	0.4409
Age at Kasai (if kasai)	(Days)	Mean ± SD	-	-	66.8 ± 28.9	53.0 ± 17.3	55.2 ± 27.0	0.157
At assessment	Age (months)	Mean ± SD	5.9 ± 1.4	4.2 ± 0.5	6.5 ± 4.1	12.2 ± 6.9	6.7 ± 2.7	**0.0032**
	Weight (Kgs)	Mean ± SD	7.2 ± 1.0	6.7 ± 0.7	6.1 ± 1.8	8.7 ± 3.7	6.6 ± 1.4	0.0821
	PELD score	score	20.7 ± 7.6	12.8 ± 7.7	12.9 ± 7.7	12.6 ± 14.3	13.5 ± 7.0	0.329
	Presence of ascites	N (%)	4 (67%)	2 (50%)	12 (67%)	3 (60%)	13 (59%)	0.9682
	Enteral nutrition support	N (%)	3 (50%)	-	10 (56%)	2 (40%)	13 (59%)	0.2739
	Parenteral nutrition support	N (%)	2 (33%)	-	6 (33%)	-	2 (9%)	0.1399
At registration on list	Age (months)	Mean ± SD	5.9 ± 1.1	4.9 ± 0.8	7.2 ± 4.4	12.4 ± 7.3	7.3 ± 2.7	**0.0264**
	Weight (Kgs)	Mean ± SD	7.2 ± 1.0	6.9 ± 0.5	6.4 ± 1.8	8.5 ± 3.9	6.6 ± 1.1	0.181
	PELD score	score	20.8 ± 8.5	13.5 ± 7.5	12.9 ± 7.7	14.4 ± 17.2	14.1 ± 7.6	0.459
Delta Peld score 1	assessment to registration on list	delta	0.2 ± 1.3	0.8 +/1 1.5	0.1 ± 0.2	1.8 ± 4.0	0.6 ± 4.3	0.618
While waiting for LT	Worsening ascites	N (%)	5 (83%)	2 (50%)	8 (44%)	2 (40%)	14 (64%)	0.4201
	Albumin infusion(s)	N (%)	5 (83%)	2 (50%)	7 (39%)	2 (40%)	16 (73%)	0.135
	Blood transfusion(s)	N (%)	4 (67%)	2 (50%)	8 (44%)	1 (20%)	12 (55%)	0.5826
	Enteral nutrition support	N (%)	3 (50%)	4 (100%)	5 (28%)	2 (40%)	9 (41%)	0.1255
	parenteral nutrition support	N (%)	4 (67%)	2 (50%)	9 (50%)	-	12 (55%)	0.212
	1 Hospital admission < 5 days	N (%)	1 (17%)	1 (25%)	5 (28%)	3 (60%)	5 (23%)	0.5118
	>1 Hospital admission < 5 days	N (%)	1 (17%)	2 (50%)	4 (22%)	1 (20%)	3 (14%)	0.574
	1 Hospital admission > 5 days	N (%)	5 (83%)	1 (25%)	7 (39%)	1 (20%)	10 (46%)	0.2195
	>1 Hospital admission > 5 days	N (%)	1 (17%)	2 (50%)	5 (28%)	-	8 (36%)	0.4159
	Recovery in ICU	N (%)	4 (67%)	1 (25%)	7 (39%)	-	6 (27%)	0.1787
At Liver transplant	Age (months)	Mean ± SD	8.1 ± 1.8	8.9 ± 0.7	13.0 ± 7.2	16.2 ± 10.1	10.9 ± 4.2	0.0511
	Weight (Kgs)	Mean ± SD	8.9 ± 1.6	8.2 ± 0.8	9.3 ± 2.6	8.5 ± 5.2	7.9 ± 1.7	0.559
	PELD score	score	22.0 ± 10.3	23.3 ± 8.3	12.8 ± 11.7	16.2 ± 16.0	21.5 ± 10.7	0.139
Clinical condition at LT	Elective-Home	N (%)	2 (33%)	2 (50%)	11 (65%)	4 (80%)	9 (41%)	0.4352
	Hospital-bound	N (%)	2 (33%)	1 (25%)	4 (24%)	1 (20%)	11 (50%)	
	ICU-bound	N (%)	2 (33%)	1 (25%)	2 (12%) *	-	2 (9%)	
Waiting time	Assessment to LT (Days)	Median (p25–p75)	43 (36.8–62)	135 (103.5–151.5)	112 (74–303)	117 (49–146)	82.5 (52.3–133.3)	0.1688
Delta PELD score 2	Assessment to LT	Mean ± SD	1.6 ± 11.4	10.5 ± 5.5	0.2 ± 11.7	3.6 ± 5.5	8.0 ± 10.8	0.163
Graft type	Full-size	N (%)	2 (33%)	3 (75%)	16 (94%)	3 (60%)	6 (27%)	**0.0202**
	Split liver graft	N (%)	2 (33%)	1 (25%)	-	2 (40%)	11 (50%)	
	Reduced liver	N (%)	-	-	-	-	2 (9%)	
	Living donor left lobe	N (%)	2 (33%)	-	1 (6%)	-	3 (14%)	
post-LT recovery	Respiratory assistance (time - days)	Median (p25–p75)	22.5 (9.3–46.3)	16.5 (10.8–44.3)	17 (5–30)	2 (1–7)	9 (5–20.3)	0.2132
	Enteral nutrition support need	N (%)	6 (100%)	4 (100%)	14 (82%)	3 (60%)	19 (91%)	0.268
	Parenteral nutrition need	N (%)	6 (100%)	3 (75%)	11 (65%)	1 (20%)	13 (59%)	0.0953
	ITU stay (days)	Median (p25–p75)	24.5 (10.5–40)	19.5 (12–46.8)	17 (8–38)	3 (3–8)	10 (6.3–19.8)	0.0802
	Hospital stay (days)	Median (p25–p75)	33 (19–54.5)	27 (24.5–70.5)	28 (19–81)	10 (9–11)	19.5 (11.3–33.3)	0.0621
Outcome	Death while waiting	N (%)	-	-	1 (6%)	-	-	0.8969
	Death after LT	N (%)	-	-	1 (6%)	-	2 (9%)	
	Alive and well	N (%)	6 (100%)	4 (100%)	16 (89%)	5 (100%)	20 (91%)	
	Current age of survivors (months)	Mean ± SD	41.6 ± 9.5	45.6 ± 17.6	37.3 ± 15.9	47.8 ± 21.1	39.8 ± 21.6	0.804

* One other child, not transplanted, was waiting in ICU and having a PELD exception score; he was removed from list because of being too sick, and died. Bold: significant values of *p*.

**Table 3:** Demographics, data and outcomes of transplanted patients according to type of donor.

			EUROPE			*p*	USA			*p*
			ALL	Living Donor LT	Deceased Donor LT		ALL	Living Donor LT	Deceased Donor LT	
N		N (%)	165	80 (48.5%)	85 (51.5%)		54	6 (11.1%)	48 (88.9%)	
Prematurity		N (%)	11 (6.7%)	7 (63.6%)	4 (36.4%)	*0.2008*	5 (9.3%)	-	5 (10.4%)	0.9339
Co-morbidity	Cardiac	N (%)	9 (5.5%)	6 (42.9%)	3 (23.1%)	*0.4810*	1 (1.9%)	-	1 (2.1%)	0.3916
	Digestive	N (%)	10 (6.1%)	5 (35.7%)	5 38.5%)		2 (3.7%)	-	2 (4.2%)	
	Other	N (%)	8 (4.8%)	3 (21.4%)	5 (38.5%)		2 (3.7%)	1 (16.7%)	1 (2.1%)	
Polysplenia Syndrome		N (%)	25 (15.2%)	11 (16.5%)	14 (16.5%)	*0.6262*	8 (14.8%)	-	8 (16.7%)	0.2786
Type of initial surgery for BA	NONE	N (%)	25 (15.2%)	13 (16.2%)	12 (14.2%)	*0.5065*	6 (11.1%)	2 (33.3%)	4 (8.3%)	0.1587
	Explorative laparotomy	N (%)	4 (2.4%)	3 (3.7%)	1 (1.2%)		4 (7.4%)	-	4 (8.3%)	
	Kasai	N (%)	136 (82.4%)	64 (80.0%)	72 (84.7%)		44 (81.5%)	4 (66.7%)	40 (83.3%)	
Kasai Centre	Non-liver expert	N (%)	44 (26.7%)	24 (37.5%)	20 (27.8%)	*0.2611*	17 (38.6%)	1 (25.0%)	16 (40.0%)	0.5321
	Liver-expert other	N (%)	12 (7.3%)	7 (10.9%)	5 (6.9%)		5 (11.4%)	-	5 (12.5%)	
	Same as LT centre	N (%)	80 (48.5%)	33 (51.6%)	47 (65.3%)		22 (50.0%)	3 (75.0%)	19 (47.5%)	
Age at Kasai (if kasai)	(months)	Mean ± SD	60.3 ± 25.0	62.2 ± 24.0	58.5 ± 25.7	*0.3826*	60.0 ± 27.2	50.0 ± 30.3	60.8 ± 27.4	0.4594
At assessment	Age (months)	Median (p25–p75)	6 (4–8)	6 (4–10)	5 (4–7)	0.0508	6.2 (4.1–7.7)	6.3 (6.2–6.5)	5.5 (4.0–7.9)	0.6095
	Weight (Kgs)	Mean ± SD	6.8 ± 2.1	6.59 ± 2.0	7.1 ± 2.0	*0.1438*	6.7 ± 1.8	6.9 ± 1.2	6.7 ± 1.9	0.8159
	PELD score	Mean ± SD	14.2 ± 8.3	14.7 ± 8.6	13.7 ± 8.0	*0.3990*	13.9 ± 8.1	18.3 ± 6.7	13.1 ± 8.3	0.1595
	Presence of ascites	N (%)	85 (51.5%)	57 (71.2%)	48 (56.5%)	**0.0486**	34 (63.0%)	5 (83.3%)	29 (60.4%)	0.2731
	Enteral nutrition support	N (%)	62 (37.6%)	25 (31.2%)	37 (43.5%)	*0.1036*	28 (51.9%)	3 (50.0%)	25 (52.1%)	0.9232
	Parenteral nutrition support	N (%)	20 (12.1%)	9 (11.2%)	11 (12.9%)	*0.7394*	10 (18.5)	-	10 (20.8)	0.2155
At registration on list	Age (months)	Median (p25–p75)	7 ± (4–10)	6 (4–8)	8 (4–11)	0.0543	6.4 (5.0–8.0)	6.8 (6.6–7.4)	6.0 (5.0–8.4)	0.448
	Weight (Kgs)	Mean ± SD	7.2 ± 2.1	6.8 ± 2.0	7.5 ± 2.2	**0.0283**	6.8 ± 1.8	6.9 ± 1.2	6.8 ± 1.8	0.9755
While waiting for LT	Worsening ascites	N (%)	120 (72.7%)	69 (86.2%)	51 (60.0%)	**0.0002**	30 (55.6%)	6 (100.0%)	24 (50.0%)	**0.0201**
	Enteral nutrition support	N (%)	66 (40%)	36 36.1%)	30 (45.0%)	*0.2496*	22 (40.7%)	2 (33.3%)	20 (47.7%)	0.6953
	Parenteral nutrition support	N (%)	37 (22.4%)	17 (21.2%)	20 (23.8%)	*0.6951*	26 (48.1%)	2 (33.3%)	24 (50.0%)	0.4411
	Albumin infusion(s)	N (%)	98 (59.4%)	56 (70.0%)	42 (50.0%)	**0.0090**	31 (57.4%)	6 (100.0%)	25 (52.1%)	**0.0252**
	Blood transfusion(s)	N (%)	48 (29.1%)	26 (32.5%)	22 (25.9%)	*0.3496*	26 (48.1%)	3 (50.0%)	23 (47.9%)	0.9233
	1 Hospital admission < 5 days	N (%)	20 (12.1%)	9 (11.2%)	11 (12.9%)	*0.7394*	15 (27.8%)	1 (16.7%)	14 (29.2%)	*0.5192*
	> 1 Hospital admission < 5 days	N (%)	21 (12.7%)	7 (8.7%)	15 (17.6%)	*0.0929*	10 (18.5%)	1 (16.7%)	9 (18.8%)	0.9014
	1 Hospital admission > 5 days	N (%)	85 (51.5%)	35 (43.7%)	50 (58.8%)	0.0528	24 (44.4%)	5 (83.3%)	19 (39.6%)	**0.042**
	> 1 Hospital admission > 5 days	N (%)	15 (9.1%)	8 (10.0%)	7 (8.2%)	*0.6935*	15 (27.8%)	1 (16.7%)	14 (29.2%)	*0.5192*
	Recovery in ICU	N (%)	22 (13.3%)	6 (7.5%)	16 (18.8%)	**0.0325**	17 (31.5%)	3 (50.0%)	14 (29.2%)	0.3002
At Liver transplant	Age (months)	Median (p25–p75)	8 (6–13)	8 (6–10)	10 (6–18)	**0.0294**	9.8 (7.6–13)	9.3 (8.3–10.5)	10.0 (7.3–14.3)	0.6297
	Weight (Kgs)	Med ± SD	8.1 ± 2.3	7.4 ± 2.0	8.7 ± 2.5	**0.0007**	8.5 ± 2.4	8.4 ± 1.7	8.6 ± 2.5	0.8853
	PELD score	Med ± SD	17.2 ± 9.8	18.8 ± 9.8	15.6 ± 9.6	**0.0395**	18.5 ± 11.8	18.2 ± 12.7	18.5 ± 11.8	0.9421
Clinical condition at LT	Elective-Home	N (%)	88 (53.3%)	47 (58.7%)	41 (48.2%)	*0.1990*	28 (51.9%)	1 (16.7%)	27 (56.2%)	0.1666
	Hospital-bound	N (%)	63 (38.2%)	29 (36.2%)	34 (40.0%)		19 (35.2%)	4 (66.7%)	15 (31.2%)	
	ICU-bound	N (%)	14 (8.5%)	4 (5.0%)	10 (11.8%)		7 (13.0%)	1 (16.7%)	6 (12.5%)	
Waiting time	Assessment to LT (Days)	Median (p25–p75)	63 (33–124)	55 (33–98)	82 (32–180)	*0.0717*	90 (49.3–148.8)	95.5 (56.3–128.8)	90.5 (49.8–152.3)	0.7726
Delta PELD score	Assessment to LT	Med ± SD	3.0 ± 6.6	4.0 ± 6.0	2.0 ± 7.0	**0.0470**	4.6 ± 10.9	-0.2 ± 15.3	5.2 ± 10.3	0.2575
Graft type	Full-size	N (%)	20 (12.1%)	-	20 (23.5%)		30 (55.6%)	-	30 (62.5%)	
	Split liver graft	N (%)	60 (36.4%)	-	60 (70.6%)		16 (29.6%)	-	16 (33.3%)	
	Reduced liver	N (%)	5 (3%)	-	5 (5.9%)		2 (3.7%)	-	2 (4.2%)	
	Living donor left lobe	N (%)	80 (48.5%)	80 (100%)	-		6 (11.1%)	6 (100.0%)	-	
LT recovery	Respiratory assistance (time - days)	Median (p25–p75)	1.0 (0–5)	0.0 (0.0–3.5)	3.0 (1.0–6.0)	**<0.0001**	10 (5–24.5)	9 (6.5–16.8)	10.5 (4.3–25.8)	0.7726
	Enteral nutrition support need	N (%)	122 (74%)	49 (61.2%)	73 (85.9%)	**0.0003**	46 (86.8%)	6 (100.0%)	40 (85.1%)	0.3103
	Parenteral nutrition need	N (%)	123 (74%)	61 (76.2%)	62 (72.9%)	*0.6258*	34 (63.0%)	5 (83.3%)	29 (60.4%)	0.2731
	ITU stay (days)	Median (p25–p75)	6 (2–12)	3.5 (2–8.5)	8.0 (4–15)	**0.0002**	12 (6.3–27.0)	10.5 (7.5–18)	12.5 (7.5–18.0)	0.9341
	Hospital stay (days)	Median (p25–p75)	26 (21–35)	24.5 (17–31.5)	27 (21–38)	**0.0312**	23 (11.3–38)	28.5 (20.5–33.5)	22.0 (11.0–39.8)	0.6794
	Death after LT	N (%)	5 (3%)	2 (2.5%)	3 (3.5%)	*0.6999*	3 (5.6%)	-	3 (6.3%)	0.529
	Alive and well	N (%)	160 (97.0%)	78 (97.5%)	82 (96.5%)		51 (94.4%)	6 (100.0%)	45 (93.8%)	

Italic and bold: significant values of *p*.

### 3.3. Center’s Waiting List Dynamics in Relation to Regional Allocation Rules

**Figure 1:**
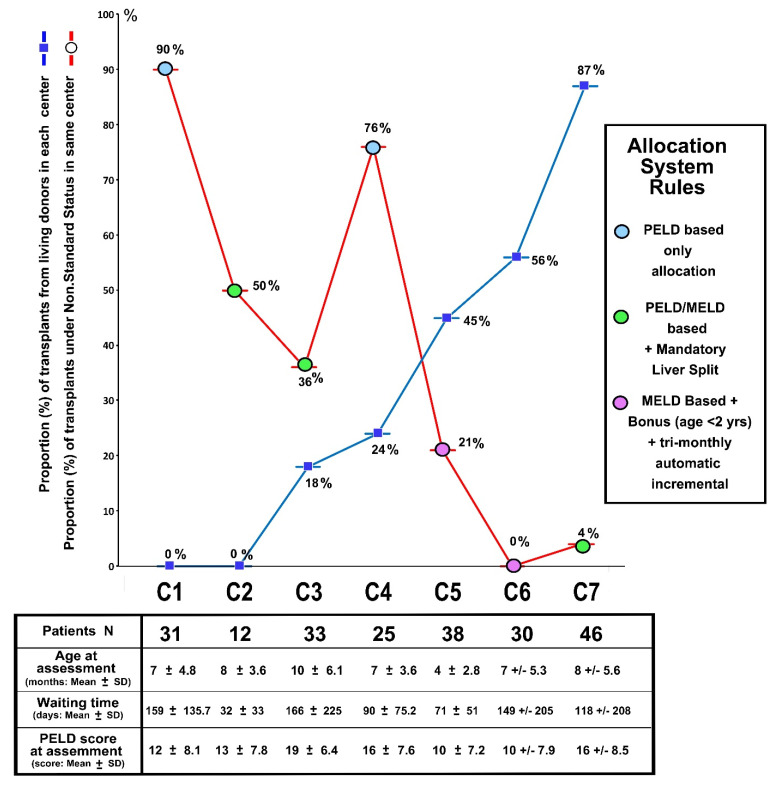
Waiting list dynamics in 7 centers participating in organ sharing/allocation system and use of PELD exception or priority status for timely transplants. Specific waiting list data from 7 separate centers (C1 to C7). 1—Age, weight and PELD score at the time of patient assessment for liver transplantation (bottom table: values given as mean ± SD) (all mean values were rounded to the nearest integer), and 2—proportion (%) of transplants performed in each center using a living related donation (blue line and dark blue spot 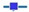) and a request for a PELD exception or similar priority score (red line) (values in % of all transplants in each center). Center organ sharing/allocation system type (see legend on the right side) are referred to as per the color spot in the figure (blue, green and purple).

**Figure 2:**
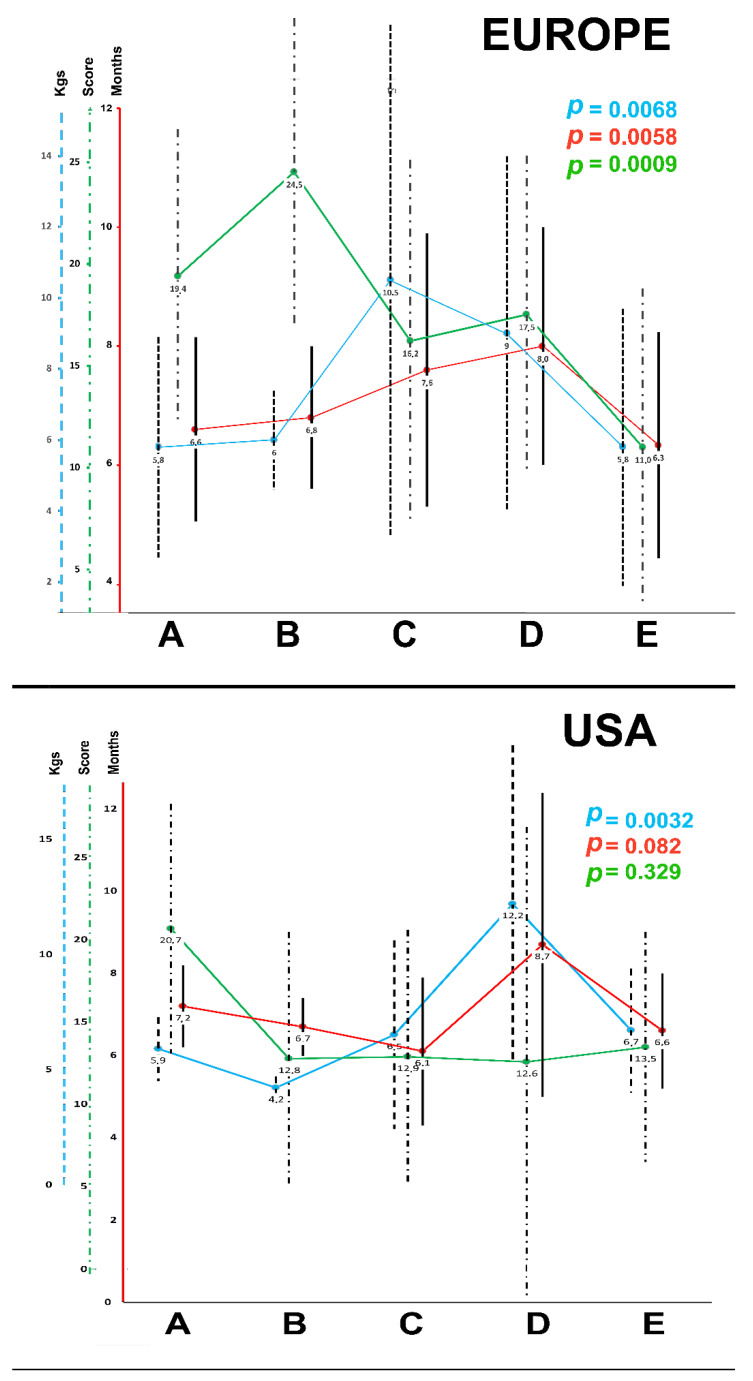
Patient characteristics at assessment for liver transplantation, as per initial management of biliary atresia. Age, weight and PELD score at assessment for liver transplantation (the dots represent the median value, with the black vertical bars representing SD): distribution per subgroups according to initial management of biliary atresia (subgroups as follow: A—no intervention and primary transplant, B—explorative laparotomy only, C—Kasai portoenterostomy in a non-specialist center, D—Kasai portoenterostomy in a specialist, but not transplant, center, and E—Kasai portoenterostomy in the transplant center).

**Figure 3:**
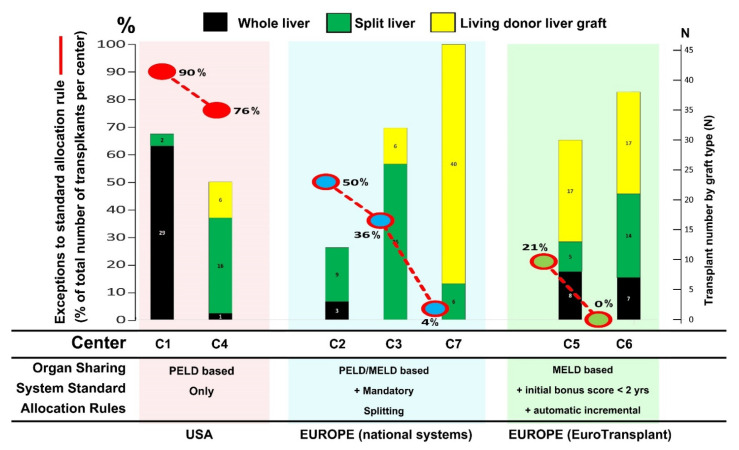
Use of PELD exception or priority status for biliary atresia patients registered on the waiting list before age 3 years, according to the type of allocation rules and types of organ used. Transplant data from 7 centers. 1—Proportion of transplants performed under PELD exception or priority status (red line) (values in % in each transplant), and 2—number of transplants and graft types used: whole livers (black), split livers (green) and living donor grafts (yellow). Centers (defined as in Figure 1) are aligned left to right according to their affiliation to one of 3 allocation systems that differ about allocation rules for small children (see legend on the bottom).

Overall, per the allocation system, the lowest use of the exception status was observed in the Eurotransplant area (centers C5 and C6—Figure 1 and Figure 2). At the center level, there was a straight correlation—proportionally to the total activity in a given center (i.e., between a high proportion of LD-LT and a very low proportion of priority requests—in that center), or the opposite, between a high proportion of whole liver grafts and a very high number of priority requests (Figure 3).

In seven centers, LT activity relied on a large national or multi-national organ exchange and allocation system that was PELD/MELD-based; although similar, rules for allocation were slightly different and three major systems were identified. All three systems had in common: (1) an emergency status for critical cases (fulminant hepatitis, urgent re-LT), (2) few priority status for special indications where PELD scores do not well-represent the condition and (3) the possibility of submitting special requests for priority status based on the critical condition of the patients as a PELD exception or similar. Differences were, however, observed between the three systems in that one also had a mandatory split approach in standard donors, and one used a variant PELD (Pediatric MELD—Eurotransplant area (https://www.eurotransplant.org, accessed on 15 March 2022)), and for children registered at age < 2 years, a bonus score at the start with an automatic score increment, every 90 days (Figure 1 and Figure 3).

Requesting some sort of priority for graft allocation because of severe clinical deterioration or complications was possible in all allocation systems—though the type of priority varied from system to system, according to the rules of the respective regional allocation system (PELD exception, bonus score points, or emergency status). In Europe, a priority status had been obtained for 29 cases (27.6%), while in the USA, a PELD exception score or an emergency status had been obtained for 47/56 cases (84%).

## 4. Discussion

A recent survey of pediatric LT in Europe over the last five decades [1] has evidenced that the outcome has steadily improved overall with time, with current results being better than ever for children in need of a LT, although the progress in the last decade is modest compared to the previous one. The former study, however, also confirmed that the youngest patients (<1 year of age at LT) continued having worse outcomes compared to the older ones, which is also suggested in previous studies [3,5,7,8,9,10,11,14,15,16,17].

Moreover, aside from the lower survival after LT, the literature provides strong evidence that those aged less than 1 year when waiting for a LT have a lower chance of benefiting from LT in time, and waiting list mortality peaks up to around 10% in this youngest group [5,6,8,11,14,15,16,17]. Prolonged waiting time is also associated with clinical deterioration, hospital boundness and/or LT under urgent conditions—all conditions associated with increased (although preventable) peri-transplant morbidity and mortality.

### 4.1. Advanced Liver Disease at Registration on Waiting List

The current study brings deeper insights into the morbidity related to the waiting period, in particular when the study evidenced that 14.1% of all patients had primary transplants (31/220 cases), and that mean age at Kasai operation was almost identical (American and European patients: 60.3 ± 25.0 days and 60.0 ± 27.2 days, respectively) (Table 1 and Table 2). This suggests that enough time was available to manage BA patients who had no chance with Kasai, or no Kasai, and planning the best timing for LT. The data demonstrate that children in both Europe and the United States were assessed for LT with already a relatively advanced stage of their liver disease, with mean (±SD) calculated PELD, at assessment, being 14.2 (±8.3) in Europe and 13.9 (±8.1) in the United States.

The proportion of children with complications at the time of their assessment was also high, including (A) the presence of clinical ascites (a marker of serious condition and poor prognosis for those awaiting LT [18,19]) in 105/165 children (63.6%) in Europe, and 31/55 cases (56%) in the United States, and (B) the need for nutrition enteral support (62/165 (37.6%) in Europe, 28/55 (51%) in the United States) or needing PN (20/165 (12%) in Europe, 10/55 (18%) in the United States). In the American patients, unlike the European ones, there were fewer differences between the live donor and deceased donor groups, except for worse ascites, greater need for albumin infusions and more hospital admissions in the live donor group (Table 3).

### 4.2. Variability of Timing in Registration on Waiting List

As the progression of biliary cirrhosis (hence of hepatic dysfunction and associated portal hypertension) is predictable when bile flow is not established, one would expect a timely referral to an LTC. The large variation in the timing for assessment (based on age at assessment in the study) is a surprising observation, with the following findings:

(1) In Europe, children who had no surgery at diagnosis were not brought to assessment at an earlier age compared to other groups. This might, however, be due to the fact that no surgery had been proposed because their diagnosis was late in life—too late for proposing a Kasai operation. In contrast, in the USA cohort, the no surgery group were listed significantly earlier than infants in the other groups. One possible reason for the delay in the surgery group is the practice of waiting three months or more to determine if bile drainage would occur in the Kasai group.

(2) There was a large variation in terms of timing of the referral for LT assessment for those who had undergone Kasai, depending on where the Kasai had been performed, particularly in the European arm of the study. Children in the European centers were younger (*p* = 0.00012) and in a better condition (as per PELD score, *p* = 0.0009) when they had been followed from diagnosis to LT within the same center (Table 1, Figure 1). In the American series, although a similar observation was found for age at assessment (*p* = 0.0032), weight and PELD score at assessment were similar in all subgroups (Table 2, Figure 1).

(3) In the 4 European patients who only had an explorative laparotomy at BA diagnosis, the mean PELD at assessment was 24.5 (SD: ±7.55), which suggests that they were referred very late, although they had no chance of cure without a LT. In contrast, the 4 US patients who only had exploratory laparotomy had a mean PELD score at assessment of 13.5 ± 7.5. These patients were evaluated for LT the earliest of all US patients (4.9 ± 0.8 months), although the numbers were too small for meaningful statistical comparisons. Interestingly, of all patients who had no Kasai (39/220, 17.7% of the cohort), all eventually did get a LT and all survived, while the actual survival of those who had the Kasai operation was 172/181 (95%) (97% and 94% actual survival after LD-LT or deceased donor LT, respectively).

(4) Overall, 31 children had primary transplants. The 2 groups (25 in Europe and 6 in USA—15% and 11% of LT, respectively) were very similar for age, weight and PELD score at assessment, and this shows that this category of patients is probably the worst as they are young, have a low weight and have the highest PELD scores in the whole series.

Overall, the trend for a variability of timing for referral was less striking in the USA cohort, suggesting that timely referral for transplant in the US is taking place from centers that do not run their own transplant programs. Nevertheless, findings in this cohort suggest that from a healthcare delivery point of view, the current situation is far from optimal, generally speaking, and is a problem to address in the future. Kohaut et al. [20], Karakoyun et al. [21] and Lampela et al. [22] had made similar observations in single-center series, showing that, in their experience, children referred for LT after Kasai performed in a different center had a poorer clinical condition and/or higher peri-transplant morbidity.

### 4.3. Burden of Care While Waiting for LT

Independently of patient death secondary to prolonged waiting time and inevitable clinical deterioration, there is a very high price to pay for those who ultimately succeed to LT and survive. This can be seen in the many aspects of child health, such as malnutrition, worsening growth retardation, recurrent infections and need for hospitalization, secondary multi-resistant bacterial colonization, neurocognitive developmental or psychomotor delay, hepatic osteodystrophy and fractures and significant psychosocial stress on both the child and the family [23,24,25,26,27].

More worrisome is the fact that not all these problems are easily or rapidly reversed after LT (i.e., bone demineralization and scoliosis, neurocognitive definitive retardation and social or scholarly integration). This data strongly suggests that reducing the waiting time and the associated clinical deterioration is a vital objective [28,29,30]. In this series, these aspects were not analyzed specifically, and only approached by looking at the need for medical support while waiting (management of worsening ascites, need for albumin or blood transfusion, hospitalizations and worsening of PELD; Table 1 and Table 2); as an example, though PN support was necessary—at referral—in 12% and 18% of cases, respectively, in European and American cohorts, these ratios increased during waiting time, to 22% and 49% of patients, respectively. Although there was also no cost assessment, another limitation of this study, it is obvious that this all translated into higher costs for the pre-transplant care, and very likely was associated with a higher cost of LT itself, as a worse condition at the time of LT is associated with longer ICU and hospital stays, respiratory assistance and need for nutritional support after LT. It is a limitation of this study, and a call for further dedicated studies [16].

Overall, this analysis and the results are a plea for earlier referral to a TC, but also a call to TC for shortening the waiting time by all means. In fact, providing all options for LT and developing more aggressive strategies for allocating liver grafts to these patients, such as splitting more livers and expanding the use of living donation, which are options that can be and should be developed further and are precisely meeting the needs for the youngest children who are most in need [31,32,33,34,35,36,37]. In the European series, the use of living donation has been important (48.5% of the series) and likely contributed to both shortening the waiting time and the excellent general outcome (97.5% actual survival over 5 years). Although the children who were proposed for living donor LT (LD-LT) were significantly younger, smaller and in a worse (PELD) condition at transplantation, they had shorter respiratory assistance and ICU/hospital stays. It confirms the important role that LD-LT can play in improving the care planning of BA children in the future, particularly in countries where the availability of deceased donor organs is limited, and LT cannot be offered in a timely manner. In countries where only a single donor option is available—LD or deceased donation—mortality on the waiting list remains high for the infants [3,5,7,8,14,38].

### 4.4. Steadily Increasing Prioritization Requests

In order to meet the needs of children who often compete with adults for organs in an environment of deceased donor shortage, one solution has been to request—and obtain—priority on the waiting list by either giving pediatric priority to organs from pediatric donors (under age 18 years), and/or by obtaining priority by bonus points or exceptions to the PELD score. The latter strategy was used in both Europe and in the USA—mostly for small infants waiting for livers whose PELD score may not accurately reflect the risk of mortality. In this study cohort, exception points were used on a surprisingly large scale, being 35% of LT, overall. Although it well-reflects that listed BA infants often rapidly deteriorate, it also suggests that graft allocation is still not adequate to this group of fragile and urgent patients.

Two very recent analyses (by SPLIT and OPTN) [39,40] confirmed that prioritization has become a necessary strategy to get infants transplanted in time. Both showed that more than half of pediatric LT in the USA are currently performed in children who received an exception score. Though the prioritization system was associated with excellent outcomes, the OPTN report showed that the proportion of PELD exception LT has steadily increased during the last decade. This strategy rapidly expanded—from 40% exception LT in 2008, to 79% in 2019—although it is not a real solution to the problem of organ shortage. Worse still, the priority allocated to one patient leaves another case a step backwards on the waiting list. This system functions as a vicious loop and the number of requests will eventually increase to become the new standard if true solutions are not implemented.

The comparison of waiting list dynamics in different allocation systems (Figure 1 and Figure 3) has evidenced that implementing a bonus score at registration for the youngest patients (<2 years of age at registration) and adding an automatic score increment every three months (Eurotransplant system) was possibly the most efficient timely allocation with the lowest number of requests for priority status. Although this strategy is an efficient redistribution of organs based on the “sickest first” concept, and also helps to increase the number of available grafts (most of the allocated donor livers would be split in this age group), it is not helpful in solving the donor shortage at the end. Surprisingly, this analysis did not evidence a major contribution of the “mandatory split” strategy, as of the three centers who benefited from that rule, two had a high proportion of priority requests (50% and 36% in centers C2 and C3, respectively—Figure 1 and Figure 3). Lastly and more interestingly, there was a good inverse correlation between a low number of special requests and a higher use of LD in a given center—with the latter observation being even clearer when comparing centers within a same allocation system (Figure 3). Altogether, this evidence calls for consideration of implementing specific allocation rules for small children at the level of the organ sharing system, and considering implementation of LD on a larger scale at the level of transplantation centers.

Many pediatric programs, both in Europe and in the USA, are still reluctant to offer split LTs on a large scale, and even more so to offer LD-LT. This situation is probably more extreme in the USA, where in 2019, whole livers were still used in around 2/3 of pediatric LT, while split and LD represented only 20.3% and 14.3%, respectively [40,41]. In Europe, whole livers, LD and splits represented approximatively one third each for pediatric LT in the last decade [1]. As split and LD are now both associated with excellent results [11,34,42,43,44,45,46], offering all the possible surgical solutions available can help to ensure timely access of children to organs and prevent the deterioration of their clinical condition or even death while on the waiting list [12]. It has been shown that the children with the highest mortality on the waiting list are those under the age of 1 year.

### 4.5. Paradigm Shift in Caring for BA and Roadmap for Future Management

In December 2021, the EASL–Lancet Liver Commission (an expert panel of health professionals from various medical disciplines, nurses and patients) called for a paradigm shift in the liver disease response in Europe (Published Online on 2 December 2021 https://doi.org/10.1016/S0140-6736(21)01701-3, accessed on 15 March 2022). After a three-year analysis, they concluded that the future health of Europeans relies on “a necessary shift in the way in which liver disease needs to be prevented, diagnosed and treated”. Their analysis confirmed that centralization of rare disease cases in multidisciplinary specialist service centers was associated with higher caseloads and, in turn, with enhanced quality of care to patients with rare diseases, such as those with primary sclerosing cholangitis and biliary atresia. The EASL–Lancet Liver Commission commented that “Early diagnosis and cost-saving therapies can be achieved by establishing effective case-finding procedures, standardized treatment protocols, and centralization of patients to high-volume pediatric liver units”.

In line with optimization of BA care, some have opted for care-centralization as the United Kingdom did in the mid-nineties. Three pediatric centers were designated for delivering a national comprehensive service for diagnosis, management and surgery of BA, including transplant services. The centralization of services and the subsequent effect on outcome was followed with attention and reported by Davenport et al. in 2004 [2], and further in 2011 [47]. Davenport et al. concluded that “*National outcome measures in BA appear better than those from previously published series from comparable countries and may be attributed to centralization of surgical and medical resources*”. In 2008, Stringer also insisted on the fact that, independently of the service type, the improvement of the general outcome is mostly dependent on timely access to transplantation [48]. More recently, centralization of BA management in Finland led to a major change and a significant increase of overall survival—from 64% to 92% [22]. Lastly, in a recent review of BA registries and outcomes, Verkade et al. [49] mentioned that the United Kingdom and Switzerland (both centralized services) had the best overall patient survival (89% and 90% at 5 years, respectively) [38,47].

Centralization of BA care is more controversial in the United States and Canada. This is perhaps in part due to the large sizes of the respective countries in contrast to European nations, but also due to the nature of pediatric surgery in America where sub-specialization among pediatric surgeons is not embraced except in very few centers. In both European and American areas, the children in Group D (who had their Kasai at one transplant center but then ultimately were transplanted at another center) were the group who were evaluated, listed and transplanted at the latest time point. Although there was no significant difference in the American series (because of the small number of patients in group D (5/55 cases)), there were significant differences in Europe for the PELD score (both at assessment and at LT), for the need for short hospitalization, or for recovery in the ICU during the waiting time. This suggests that LTs in children should be concentrated in liver-specialized pediatric units who can seamlessly take care of an infant from diagnosis of BA to the performance of LT.

As an alternative to centralization, improving integration and collaboration between non-LTC and LTC could be a solution and optimization of the current situation. It would imply a set of innovative strategies or optimizations. Focusing on substantially earlier recognition of Kasai failure, earlier contacts to the LTC for sharing information and optimizing timing for referral, sharing post-Kasai and LT protocols may improve delayed adequate healthcare delivery and lead to earlier referral and listing. These proposals have already been made by various authors but have not been implemented: they could be the cornerstone of a roadmap for improving the management of BA children in the future [50,51,52,53,54,55,56]. Additionally, as the data evidence that children who did not undergo the Kasai were referred and transplanted earlier in both European and American cohorts, part of the solution could be to define parameters (other than presentation at age > 120 days or significant portal hypertension) that allow predicting success, or not, with the Kasai operation. Schneider et al., for example, suggested that failure to normalize serum bilirubin within three months after Kasai should elicit prompt evaluation for LT as extended native liver survival is exceptional among these patients [50]. Moreover, though a “Kasai success predicting score” remains far from reality, other groups support more research in that direction as part of BA management—a strategy that would allow early primary transplantation [47,54,57,58,59,60,61].

### 4.6. More than Ever, Room for Technical Solutions

The zero mortality in Europe during the waiting time is remarkable and may reflect the larger use of LD and split liver grafts in Europe (48.5%). Pre-transplant mortality in America is also low, possibly more a reflection of the greater access to pediatric organs (54% of LT were with full-size livers in the American cohort, versus 12% of cases in Europe)—even though the latter access is not present in all geographic American areas [48]. Though the very low waiting list mortality in this cohort could suggest that allocation systems are efficient and sufficient, the frequent need for an exception status (especially in America) and the high demand for medical support before LT evidence that access to LT can and must be improved worldwide. The best approach for an expert pediatric LTC is to combine offering all possible transplant modalities, i.e., implementing split as a standard procedure in every optimal multi-organ donor and considering a living donor program [11,12,34,43,44,45,46,62,63,64]. Since deceased donor organs are a limited resource, and because not all recipients can benefit from a LD for their LT, the combination of both approaches is in fact synergistic and strategic—the most highly probable manner for a single LTC to meet the needs of all their patients in a timely fashion [1,6,11,21,31,35,42,47].

Major differences between the European and the American cohorts are the use of living donors and split LTs. The European study reports an almost 50–50 division in the use of living and deceased donor transplants. In the deceased donor group, 70% of grafts are split LTs, and only 20 of 165 transplants are whole livers. In contrast, the American group had only 11% (6/54) utilization of live donors and only 16/54 (30%) of the rest were split livers. A surprising 30 of 54 LTs utilized whole organs. This difference in utilization of graft types and donor types is more a function of the disparity between the two American centers than it is between Europe and America; while not analyzed here, the vast majority of the whole LTs came from one center, and the majority of the live donors and split LTs from the other center [46,64,65]. This highlights not only different practices between Europe and North America, but between regions in the United States. European centers also differed, as two main allocation rule types were identified; interestingly, it was associated with differences in terms of transplant practice and the need for requesting exceptional status (Figure 3). This deserves further studies.

### 4.7. Study Limits and Strengths

The observations and results in this study are subject to some limitations. First, the study was retrospective. Due to its observational-only character, it was not adjusted for other elements that may have played a role in a timely referral of patients to the transplant center (i.e., socio-familial issues, intercurrent infections), nor in the timing of LT (i.e., local policies for LD, graft allocation rules). Second, the study was a joint venture between centers who were members of the BARD association and partners in the European reference network on pediatric hepatological diseases (The study is part of a European reference network for the pediatric hepatological diseases (ERN RARE-LIVER) initiative, and was promoted by the BARD association (www.bard-online.com, accessed on 15 March 2022). All European center authors are partners of the ERN RARE-LIVER). Due to their special interest in managing BA children, and their expertise, the results may be not representative of those of the main transplant network.

Despite the limitations, the analysis also has several strengths. Firstly, despite important differences between Europe and the USA, in terms of both organization of the health system and allocation of liver grafts to children, very similar findings were evidenced. Secondly, the study was limited to only the last five years of managing BA children, thus reflecting the current medical practice very well, and the cohort was large (220 BA patients) enough to eventually represent the management of BA children in both Europe and the USA.

## 5. Conclusions

Developing improved healthcare solutions not only imposes an obligation for scrupulous and frequent analysis of clinical practice and standards, but also relies on auditing clinical outcomes—a systemic reflective practice. However, if this reflection does not result in bringing new skills and improving the knowledge of the practitioners, nor in convincing health providers and healthcare administrators to implement changes that will result in new practices, it is unlikely that patients will benefit and eventually get better care.

In managing BA—a disease that was associated with close to a 100% death rate until five decades ago—both the Kasai portoenterostomy and LT have been instrumental in allowing a continuously growing number of children to survive. As LT currently results in BA patient survival exceeding 95% in many expert LT centers, the paradigm for BA management optimization and survival has now shifted overall to the pre-LT management [6].

Evidence has now accumulated that demonstrates that BA management can only be improved by either the centralization of care (as already performed or proposed for some other rare conditions in a few countries [66,67,68,69]), or the creation of networks dedicated to the timely referral to a pediatric transplant center of excellence. As a new way of thinking and because a large proportion of BA will eventually come to LT, post-Kasai care should be aimed at identifying children in need of LT. Standard Kasai follow-up should be a time not only for pediatric hepatologists to monitor the progress of the baby, but also for involving pediatric transplant surgeons more closely than in the past.

Pre-emptive LT assessment, early listing and timely transplant are likely the next necessary steps to further improve the general outcome. In a non-centralized system as seen in the USA, cooperation between expert centers and other tertiary hospitals is also essential to deliver the strategies of caring for BA children who are potential candidates for LT, and ensuring the necessary medical and surgical support to offer a timely transplant.

Lastly, this series suggests that both mandatory liver splitting policy and LD may play an important role in the immediate future to offer LT in a timely manner to all BA candidates in need, and in particular for the younger ones.

## Abbreviations

ERN RARE-LIVER	European reference network for pediatric hepatological diseases.
BARD	Biliary Atresia and Related Diseases (multi-purpose online platform for study of biliary atresia and related diseases: http://www.bard-online.com, accessed on 10 March 2022)
BA	Biliary atresia
LT	Liver transplantation
PELD	Pediatric End-Stage Liver score
ICU	Intensive care unit
LD	Living donor
LTC	Pediatric liver transplant center
PN	Parenteral nutrition
